# Book Reviews

**Published:** 1885-04

**Authors:** 


### BOOK REVIEWS.

THE ELEMENTS OF PHYSIOLOGICAL PHYSICS. *By* J. MCGREGOR-ROBERTSON, M. A., M. B., C. M., *Muirhead Demonstrator of Physiology, and Assistant to the Professor of Physiology in the University of Glasgow. Illustrated with 219 Engravings on Wood. 16 mo, Cloth, pp. xv—528. Philadelphia:* H. C. LEA'S SON & CO. 1884. *Chicago:* JANSEN, MCCLURG & CO.

Physiology is becoming every day more practical and exact. The time may come when it will favorably compare with mechanics and chemistry in this respect, and this work will contribute to such advancement. Although it is issued in the series of "Manuals for Students of Medicine," there are certainly but few practitioners who would not derive some benefit from its careful perusal. It is true to its title, terse and clear. Its make up is English, and its illustrations very creditable. It is especially adapted to those medical men who did not have the advantage of studying the larger works on Natural Philosophy by Ganot and Deschanel. Part I. deals with Electricity and Magnetism. It is exhaustive, and even enters into the therapeutics of electricity; though it fails in pointing to the origin of animal electric currents, and the organic production of electricity, as observed in torpedo fishes and other animals.

Part II. treats of the Myograph; Part III. of Hydraulics, giving the mechanics of the circulation, *i.e.*, Osmosis, and the Sphygmograph, etc. The subject of Pneumatics follows. The subject of Optics is dealt with at considerable length, and includes the principles of the Polariscope, Ophthalmoscope,

Microscope, Spectroscope, etc. Acoustics is treated of in Part VI.; Heat in Part VII., and Dynamics in Part VIII. It will thus appear that the ordinary order of these subjects has been reversed and partly mixed, without any particular advantage resulting therefrom. The philosophy of the subject is rather meagre, and the abundant suggestions to be drawn from comparative physiology in regard to the subject-matter are still to come. H. D. V.

---

A MANUAL OF THE MEDICAL BOTANY OF NORTH AMERICA. *By* LAWRENCE JOHNSON, A. M., M. D., *Lecturer on Medical Botany, Med. Dep. University of the City of New York; Member of the Committee of Revision of the Pharmacopœia, etc. 8vo., cloth, pp. xv—292. New York:* WILLIAM WOOD & CO. 1884. *Chicago:* W. T. KEENER.

This is one of the valuable books issued in Wood's Library. Its make up is an improvement on many other books in the series, and the profuse illustrations which adorn it are elegant and true to nature. The nine plates drawn from photographs are admirable, but liable to be spoiled by the carelessness of the printer in omitting to face them with silk paper sheets.

This work, which might have gained by separate publication, will prove a delight to medical and pharmaceutical students in their rambling after botanical specimens, whether tramping the Green Mountains and the Adirondacks, or the iridescent prairies of the West. Part I., or the Introduction to the rest of the volume, is a short sketch of Botany, which would have gained by an addition to it of a key of classification, such as is found in Gray's. As it is, it will nevertheless be of much use to those not familiar with botany. A glossary ends this introduction. The order followed in the rest of the volume is the old one, that is, the reverse of that found in Sachs, and in C. E. Bessey. The aggregation of orders into cohorts is omitted, though doubtless of great use, especially to beginners, and also to students of biology. Each of the 500 plants treated of is referred to six headings—description, habitat, part used, constituents, preparations, medical properties and uses. The description is complete for each order, and will enable any one to recognize the plant very readily in the field.

H. D. V.

---

HOOPER'S PHYSICIAN'S VADE MECUM: A MANUAL OF THE PRINCIPLES AND PRACTICE OF PHYSICS, WITH AN OUTLINE OF GENERAL PATHOLOGY, THERAPEUTICS AND HYGIENE. Tenth Edition. *Revised by* W. A. GUY, M. B., F. R. S., *and* JOHN HARLEY, M. B., F. L. S. *8 vo, cloth, pp 358. Vol. II. Wood's Library. 1884.*

There is such a thing as stealing property which proves more cumbersome than valuable afterward, or which brings derision to its possessor, as the monk's medals to Gil Blas; but whether the non-existent international copyright was taken *disadvantage* of or not in this case, the book is highly interesting for its blunders. American students are often provoked by the infusions and decoctions advocated in Ziemssen's Cyclopædia, but the British nostrums far exceed these in retrogressive progress. The hypodermic use of sulphate of morphia is spoken of thus: "The subcutaneous use of morphia is often a dangerous proceeding, and the first dose by this method should never exceed one-eighth of a grain, and it is well when we are unacquainted with the constitution of the patient to introduce one-sixtieth grain of atropia previously." The formulæ for pills very frequently call for five gr. pills, which any student here would prefer to have put up in capsules. But these are probably unknown to the writers, just as fluid extracts seem to be. The following is a sample of the treatment of various diseases, and is recommended in cases of acute gastritis: (Please compare with Bartholow.) I. "Six to twelve leeches to the pit of the stomach, and iced water or ice, externally and internally. Free action of the bowels in the absence of diarrhœa, by the use of emollient clysters. The free and frequent use of mucilaginous diluents, such as gruel, linseed tea, or barley-water.

II. "The sickness, restlessness, and pain, are best relieved by soda water and small doses of dilute hydrocyanic acid (m iii to m v) combined with opium." Nothing is said of the most important symptom, the vomiting.

It is time that more attention should be given to valuable literary efforts at home, and that a duty be established on the importation of foreign rags. H. D. V.

